# Evaluation of the OPTESK VR training simulator for gastric bleeding treatment

**DOI:** 10.1186/s42234-026-00216-w

**Published:** 2026-10-19

**Authors:** Annika Köhler, Irshath Ali Syed, Jana Sophia Theile, Ioannis Kafetzis, Joel Troya, Jan-Peter Grunz, Ulrike W. Denzer, Ivica Grgic, Benjamin M. Walter, Rüdiger Pryss, Stavros Dimitriadis, Alexander Hann

**Affiliations:** 1 Interventional and Experimental Endoscopy (InExEn), Department of Internal Medicine II, University Hospital Würzburg, Würzburg, Germany; 2 Bavarian Center for Cancer Research (BZKF), Würzburg, Germany; 3 Department of Diagnostic and Interventional Radiology, University Hospital Würzburg, Würzburg, Germany; 4 Division of Gastroenterology and Endocrinology, Centre for Internal Medicine, Philipps University of Marburg & University Hospital Marburg, Marburg, Germany; 5 Department of Internal Medicine and Nephrology, Marburg University, University Hospital Giessen and Marburg, Marburg, Germany; 6 Institute for Artificial Intelligence in Medicine, Marburg University, University Hospital Giessen and Marburg, Marburg, Germany; 7 Department of Internal Medicine I, University Hospital Ulm, Ulm, Germany; 8 Endoscopy Research Group, University Hospital Ulm, Ulm, Germany; 9 Institute of Clinical Epidemiology and Biometry, University of Würzburg, Würzburg, Germany; 10 Institute of Medical Data Science, University Hospital Würzburg, Würzburg, Germany; 11 Private gastroenterology practice, Egnatia 106, 54622 Thessaloniki, Greece

**Keywords:** Virtual reality, Gastroscopy, Upper gastrointestinal bleeding, Gastroenterology, Endoscopy training, Medical education, Simulator training

## Abstract

**Background:**

Bleeding in the upper gastrointestinal tract is a common and potentially life-threatening emergency that requires rapid and highly precise endoscopic intervention. As supervised hands-on training is limited and often restricted to living animal models, an immersive virtual reality (VR) simulation offers a risk-free and accessible alternative.

**Methods:**

A novel open-source VR training simulator, OPTESK, was developed for the treatment of upper gastrointestinal (GI) bleeding. The system consists of a combination of conventional endoscopes used in routine clinical practice, which can be operated in conjunction with commercially available head-mounted displays and the in-house developed OPTESK hardware. The simulator covers two scenarios: the treatment of a non-bleeding angiodysplasia with argon plasma coagulation (APC) and the treatment of a bleeding angiodysplasia starting with the injection of adrenaline followed by APC therapy. The simulator was evaluated in a multicenter user study in terms of workload, usability, immersion, cybersickness, and face, content, and construct validity. User performance was assessed via scores on time, amount of treated area, and correctly applied APC intensity.

**Results:**

In total, 44 participants from more than 20 centers were included in the study. Successful hemostatic control in the second scenario was achieved by 97.4% of the participants. Regarding the content validity, participants rated the helpfulness of the simulator for training the management of bleeding with a median of 4 on a scale from 1 (strongly disagree) to 5 (strongly agree). Mean perceived workload (NASA-TLX) and usability (SUS) were rated with 34.2/100 and 69.6/100 respectively. Face validity questions regarding the realism of endoscope movement, stomach anatomy, and application of APC were further rated with a median of 4 on the scale from 1 to 5. Regarding construct validity, gastroscopy experience showed significantly faster treatment of bleeding related tasks for both scenario one (*p* = .008) and two (*p* = .011) compared to participants with less gastroscopy experience.

**Conclusions:**

OPTESK can serve as a valuable training tool for endoscopic treatment of bleeding in the upper GI tract. This approach may help reduce dependence on animal-based training and enable risk-free practice prior to clinical intervention.

**Supplementary Information:**

The online version contains supplementary material available at https://doi.org/10.1186/s42234-026-00216-w.

## Background

Upper gastrointestinal bleeding (UGIB) is a life-threatening condition whose incidence is rising due to the increasing use of oral anticoagulants (Götz et al. 2025). The current treatment standard of UGIB is endoscopy, that can diagnose and treat bleeding in the same session. Management of UGIB is mainly performed using endoscopic clips, argon plasma coagulation (APC), injection therapy using diluted adrenaline, and hemostatic powder (Götz et al. 2025; Gralnek et al. 2021; Shung and Laine 2024). Training in clinical practice ensures high examination quality and is crucial for maintaining patient safety. For this reason, guidelines and structured training for the optimal performance of endoscopic procedures are a major focus of ongoing research (Choy et al. 2020; Antonelli et al. 2024). Currently, endoscopists are mainly trained for endoscopic treatment procedures either directly on patients or on living animal models. This results in limited training options, raises ethical concerns, and is associated with high costs (Coluccio et al. 2025; Finocchiaro et al. 2021).

Simulators provide an effective training modality, allowing complex procedures to be performed in a controlled, risk-free environment and are suggested to be used in the training phase of novices (Antonelli et al. 2024). Endoscopic training can be performed via either mechanical simulators enabling the use of commercially available endoscopes (Fujii et al. 2021; Olabintan et al. 2026; Kanno et al. 2024), ex vivo or in vivo animal models (Coluccio et al. 2025), computer-based (Finocchiaro et al. 2021; Erden et al. 2024; Hohlstein et al. 2026), or virtual reality (VR) applications (Henniger et al. 2024). Computer-based simulators present the virtual training environment on conventional computer screens, whereas VR systems use head-mounted displays (HMDs) engaging visual and auditory senses to immerse the user in the simulated environment. Each modality offers unique strengths and limitations in terms of realism, costs, ethical concerns, and haptic feedback (Coluccio et al. 2025; Finocchiaro et al. 2021). The use of VR is increasingly researched and utilized in clinical settings, particularly in the field of diagnostics through enabling a different spatial-anatomical understanding (Zolkin et al. 2026; Eisenträger et al. 2026). VR simulators can further provide a realistic medical environment, including a complete set of sensory stimuli, as found in the real-world setting (Mao et al. 2021; Jensen and Konradsen 2018). Increasing the realism of a VR simulator by adding realistic distractions (Andersen et al. 2023), haptic, audio, visual, and performance feedback, as well as different levels of difficulty (Mao et al. 2021; Jensen and Konradsen 2018; Bogar et al. 2024), can enhance the training quality compared to non-VR simulators (Jensen and Konradsen 2018; Andersen et al. 2023) and can lead to an improved knowledge gain through three-dimensional (3D) perception in full immersion (Gutiérrez et al. 2007). VR simulators can also increase the learning experience (Jensen and Konradsen 2018; Bogar et al. 2024), do not require supervision (Bogar et al. 2024), and can be more cost efficient (Mao et al. 2021) than mechanical or computer-based simulators. As a limitation, VR can lack haptic feedback based on the use case (Mao et al. 2021; Bogar et al. 2024) and can induce symptoms of cybersickness (Jensen and Konradsen 2018).

VR simulators are currently most common for surgical applications (Mao et al. 2021), such as laparoscopic procedures, demonstrating comparable training effectiveness with mechanical simulators (Bogar et al. 2024). In the field of endoscopy, computer-based simulators enable training of diverse procedures such as UGIB treatment (Coluccio et al. 2025; Finocchiaro et al. 2021), bariatric interventions including application of APC (Erden et al. 2024), and resection treatment with endoscopic submucosal dissection (ESD) marking (Hohlstein et al. 2026). For some of these simulators, custom-built training endoscopes were developed (Erden et al. 2024; Hohlstein et al. 2026). Additionally, VR has been used to train physicians and nurses for the endoscopic procedure including peri-interventional tasks (Henniger et al. 2024). To the best of our knowledge, it has not yet been possible to use commercially available endoscopes with HMD-based VR simulators. Additionally, there was no evaluation of a VR training simulator for the endoscopic treatment of UGIB.

This work introduces the open platform for animal material-free endoscopic simulation and complication management (in German: **O**ffene **P**lattform zur **T**iermaterialfreien **E**ndoskopischen **S**imulation und **K**omplikationsmanagement), OPTESK, an open-source VR training simulator for the treatment of UGIB. OPTESK uses and is compatible with gastroscopes from clinical routine, as a controller to improve haptic feedback and immersion. Sensors attached to the endoscope detect its movements and translate them into movements of the virtual endoscope experienced by the trainee via the HMD. In this study, involving physicians from multiple centers, we evaluated the workload, usability, immersion, cybersickness, and face, content, and construct validity of OPTESK. User performance was assessed via scores on time, amount of treated area, and correctly applied APC intensity.

## Methods

### OPTESK simulator

**Fig. 1:**
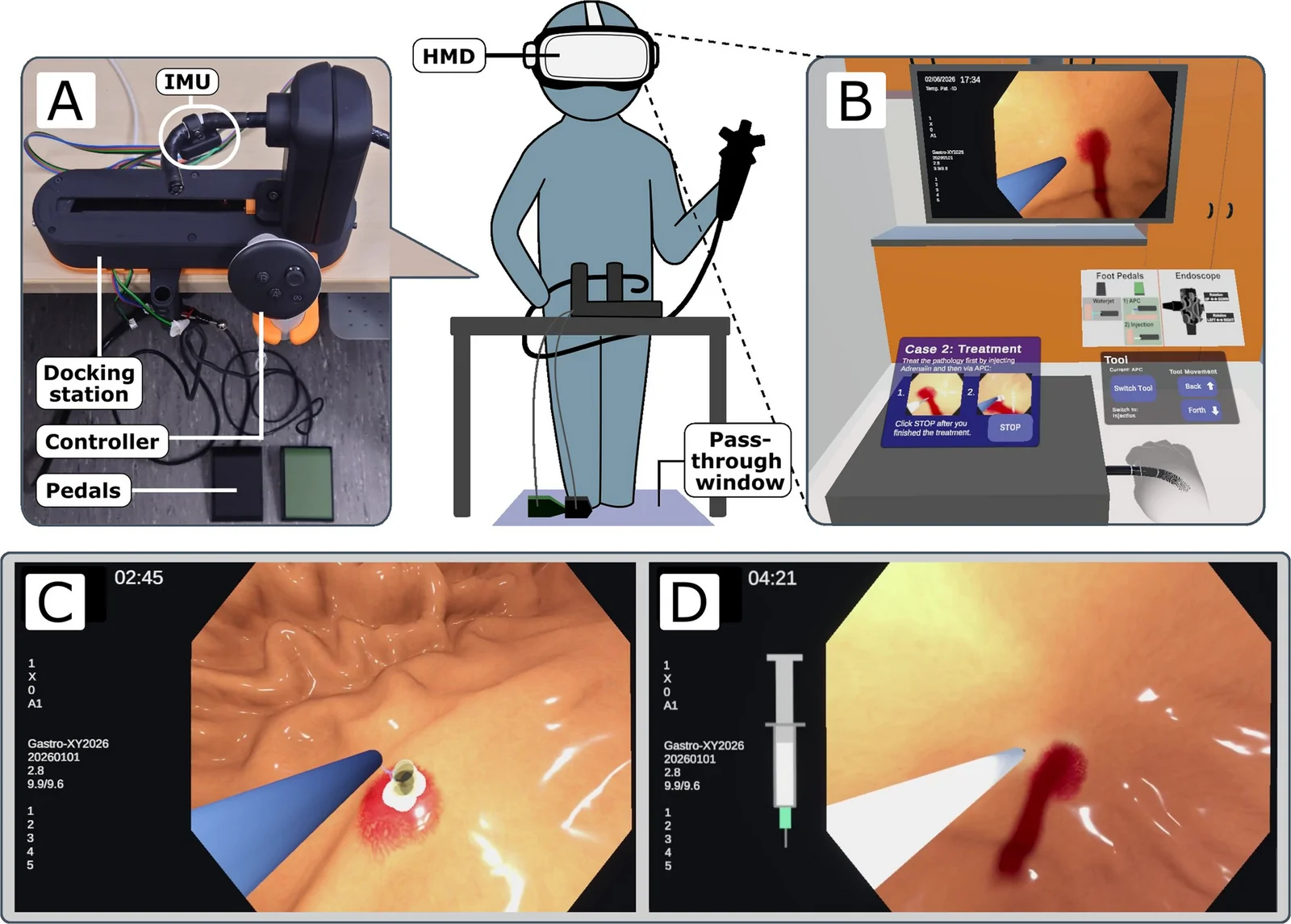
Overview of the OPTESK simulator setup. The gastroscope is attached to the 3D printed docking station containing the sensor assembly and the two pedals for controlling instruments during the simulation. Pedals are made visible to the user during the simulation via a passthrough window, indicated with a blue area on the floor. **A** Closeup of the hardware used for the simulator. The custom-built docking station of the simulator, with the endoscope mounted on it. Attached on the tip of the endoscope is the Inertial Measurement Unit sensor used to track the bending. The foot pedals used in the simulator are placed on the floor. **B** View of the virtual endoscopy room from within the head-mounted display. **C** Virtual endoscopy screen footage of applying argon plasma coagulation within the stomach. **D** Virtual endoscopy screen footage of injecting adrenaline within the stomach. IMU: Inertial Measurement Unit; HMD: Head-Mounted Display

The novel VR simulator OPTESK was developed to train the treatment of bleeding in the upper GI tract. The simulator allows the usage of any commercially available gastroscope. The gastroscope and custom-built foot pedals with in-built push buttons act as controllers for the simulation. Using a commercially available gastroscope, users have the tactile experience of operating a real endoscope including its inherent mechanical resistance from turning the wheels and from pushing and pulling the tube docked at the 3D printed station. Scenarios of the OPTESK simulator include a non-bleeding and bleeding angiodysplasia which are treated with injection therapy with diluted adrenalin and APC (Fig. 1). For running the simulation, the Meta Quest 3 (Meta Platforms, Inc., Menlo Park, California, United States) was used. The Quest 3 was connected to a gaming laptop (CPU: AMD Ryzen 9 8940HX (AMD, Santa Clara, United States of America), GPU: NVIDIA GeForce RTX 5070 (NVIDIA, Santa Clara, United States of America)), which also received rotational and positional data from the sensors connected to the real gastroscope via a microcontroller (Additional file 1—Sup. Figure 1). The microcontroller further received data from the push buttons of the foot pedals to trigger instrument usage during the simulation. In the simulation, the gastroscope was displayed via a 3D modeled virtual representation of the endoscope’s control section and the tip of the insertion tube, which mirrored the bending of the endoscope in the real world. The left Meta Quest 3 controller was attached to the control section of the real endoscope to track its position and move the 3D model accordingly within the virtual environment. The tip of the 3D modelled insertion tube was anchored to the virtual counterpart of the 3D printed docking station. The right Meta Quest 3 controller was attached to the docking station positioning the entire virtual environment to its corresponding setup in reality. The pedals were visible to the user via a passthrough window on the floor of the virtual endoscopy room. A passthrough window acts as a virtual screen in the simulation, which displays a view of the real world captured by the headset’s cameras in real-time. For the development of the simulator Unity Pro 6.3 (Unity Technologies, San Francisco, California, United States) was used. Additional file 1 contains a more detailed explanation of the hardware setup. The complete hardware setup including 3D printing files, schematics of the wiring, and the software can be downloaded for training and research purposes (https://share.ukw.de/index.php/s/3ttXgJajntqcDiY).

### Study design

The validity and usability of OPTESK were evaluated via a user study conducted at two national German conferences. These conferences included the Congress of the German Society for Endoscopy and Imaging Techniques (DGE-BV, March 25–27, 2026) and the Congress of the German Society of Internal Medicine (DGIM, April 18–21, 2026). Participants were recruited on a voluntary basis and informed about the procedure and general purpose of the study. Only participants with a specialty in gastroenterology, internal medicine, or surgery with endoscopy experience were included in the final analysis.

After signing the informed consent, participants filled out a pre-questionnaire regarding demographics, their general opinion on simulator usage based on previous work (Hohlstein et al. 2026), and their VR experience. Participants subsequently completed a tutorial on the simulator to become familiar with the VR environment (Schaal et al. 2026). The tutorial started with application of APC to a specific area of the stomach wall, throughout which participants received feedback on their performance represented by the percentage of treated area and treatment intensity. Subsequently, the tutorial focused on the injection of adrenaline at a specific point of the stomach and receiving feedback on its successful application.

After the tutorials, the user started with task one: treating a non-bleeding angiodysplasia via APC, followed by task two: treating a bleeding angiodysplasia by injecting diluted adrenaline prior to applying APC to achieve hemostatic control. Participants received feedback on the percentage of treated area, APC intensity, and time durations after each task. Finally, participants were asked to fill out a post-questionnaire.

The questionnaire included items assessing perceived workload and usability, evaluated via the standardized questionnaires of the raw NASA Task Load Index (NASA-TLX) (Hart et al. 1988) and the System Usability Scale (SUS) (Brooke 1996). Participants further assessed immersion, face and content validity (5-point Likert scale: 1 strongly disagree, 5 strongly agree), cybersickness (1 none, 5 severe), and could optionally give free-text feedback. The non-standardized questions were adopted from previous works (Erden et al. 2024; Henniger et al. 2024; Kennedy et al. 1993; Bouchard et al. 2004) and can be seen in Additional file 2. Task durations, percentages of treated angiodysplasia area and correctly applied APC intensity, as well as the achievement of hemostatic control were recorded by the system. The APC treatment intensity was calculated based on the percentage of angiodysplasia area that shows a brown coloration, ranging from white to black, after the application of APC.

Subgroup analyses regarding participants’ gender (females, males), age (≤ 40, > 40), self-reported immersion (low immersion ≤ 3, high immersion > 3), VR-experience (no VR-experience = 1, VR-experience > 1), and gastroscopy-experience (less experienced vs experienced gastroscopists, ≤ 500 vs > 500 gastroscopies) were performed.

### Statistical analysis

Descriptive statistics for Likert questions were reported as medians with first and third quartiles (Q1-Q3). The NASA-TLX and SUS scores were calculated based on their standardized guidelines (Hart et al. 1988; Brooke 1996) and together with task durations reported via means (M) and standard deviations (SD). The scores regarding the angiodysplasia area treated and correct intensity of APC were recorded in percentages and reported via M and SD. For the subgroup analysis, the non-parametric Mann–Whitney U test was applied for all Likert-scale data. For continuous data (NASA-TLX and its subscales, SUS, task time, and performance scores), normal distribution was assessed using the Shapiro–Wilk test and the homogeneity of variances using Levene’s test. In case of normally distributed data, independent-samples t-tests were used when homogeneity of variances was met and Welch’s t-test when variances were unequal. For non-normally distributed data, Mann–Whitney U tests were conducted. Free-text feedback was analyzed using an inductive content analysis (Mayring 2021). In cases of missing data, a pairwise deletion approach was applied. Participants with incomplete data were excluded only from analyses requiring these missing values. The statistical analysis was conducted using R version 4.4.3 in RStudio (Posit Software, Boston, Massachusetts, United States). A *p*-value < 0.05 was considered statistically significant.

### Ethics

The medical ethics committee at the Julius Maximilian University of Würzburg waived the study’s requirement for formal ethical approval (2026–36-ka). For the user study no personal identifiable information was recorded and all participants provided informed consent prior to participation. The principles of the Declaration of Helsinki and its subsequent amendments were followed for the conduction of the study.

## Results

### Study participants

A total of 44 physicians from over 20 centers agreed to participate in the study (Table 1) and were included in the analysis of the data. In case of missing data, participants were only excluded from specific analyses, as further explained in Additional file 3. The highest number of exclusions occurred for task two, for which five participants were excluded due to missing data. The descriptive statistics revealed an imbalanced gender distribution, with 68.2% males and 31.8% females. Over half of the participants were experts (56.8%), with over 1000 performed gastroscopies and the majority were gastroenterologists (86.4%). The participants’ general attitude towards simulator training from the pre-questionnaire showed that 84% agree on the implementation of simulator training in endoscopy training, while 55% believe this should be mandatory (Additional file 4—Sup. Figure 1).

**Table 1:** Descriptive statistics of participant characteristics, general attitudes towards simulators in endoscopy training, and VR experience

Characteristic	Value
Gender, n (%)	
Male	30 (68.2%)
Female	14 (31.8%)
Age, median (Q1—Q3)	41 (36.8–50.2)
Professional Title, n (%)	
Resident	9 (20.5%)
Senior Physician	2 (4.5%)
Specialist	33 (75.0%)
Specialty, n (%)	
Gastroenterology	38 (86.4%)
Internal Medicine	4 (9.1%)
Surgery	2 (4.5%)
Gastroscopies Performed, n (%)	
< 250	9 (20.5%)
250—500	2 (4.5%)
500—1000	8 (18.2%)
> 1000	25 (56.8%)
General Attitude Towards Simulators, median (Q1—Q3)	
Simulator training in general (any simulator) should be implemented in endoscopy training	5 (4—5)
Simulator training (any simulator) should be mandatory before starting endoscopy in patients	4 (2—5)
Previous VR-Experience, median (Q1—Q3)	2 (1—4)

### Content and face validity

**Fig. 2:**
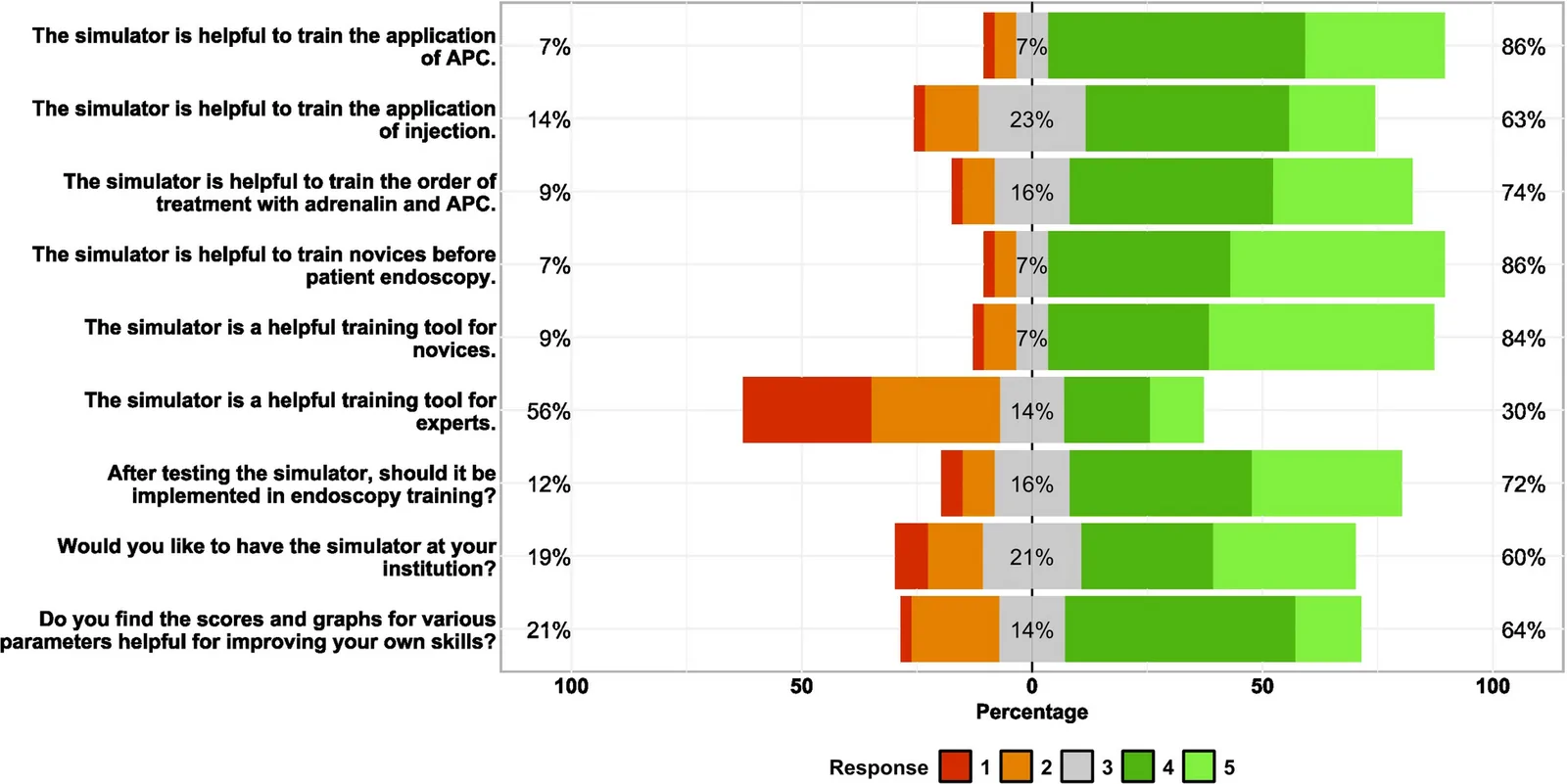
Likert plot of the content validity questions. Participants rated the questions on a 5-Point Likert Scale ranging from strongly disagree (1) to strongly agree (5)

Overall content validity questions were rated with a median of 4 on a scale of 1 (strongly disagree) to 5 (strongly agree), except for the simulator’s helpfulness to train experts with a median of 2 with the first and third quartiles at Q1-Q3 = 1–4 (Fig. 2). Participants agreed (median = 4) that OPTESK is helpful to train the application of APC, injection, and their sequence. Participants further agreed (median = 4) that the simulator is helpful for training novices, should be included in endoscopy training, and is helpful for training prior to first patient intervention. Participants also agreed (median = 4) that the scores provided by the OPTESK simulator can help to improve their own skills and they welcomed the adoption of OPTESK at their own institution.

Median responses for the face validity questions ranged from 3 to 4 on a scale of 1 (strongly disagree) to 5 (strongly agree) (Additional file 4—Sup. Figure 2). Participants rated the VR simulator’s virtual gastroscope movement, the visualization and application of APC, the instruments, the stomach anatomy, and the endoscopy screen footage as most realistic with a median of 4. Participants rated the injected areas and the application of injection as least realistic with a median of 3.

### Construct validity

**Fig. 3:**
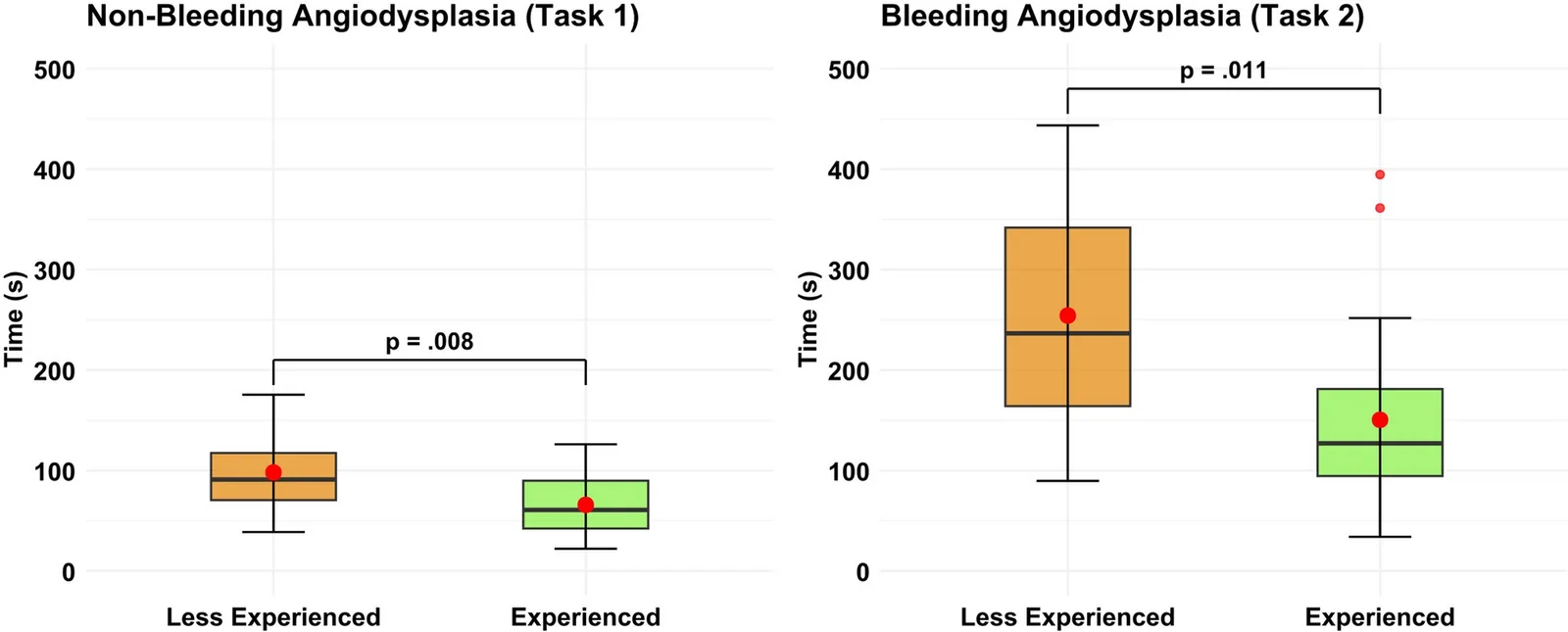
Box-and-whisker plots illustrating treatment durations in seconds for task one (left) and task two (right) by less experienced (*n* = 11) and experienced endoscopists (*n* = 33). Whiskers denote the 1.5 × interquartile range and points beyond the whiskers are plotted as outliers

The mean time to complete task one was 74.0 s ± 35.5 s, with 72.4% ± 20.6% treated angiodysplasia area, and 34.5% ± 14.5% area of correctly applied APC intensity. The mean time of task two, treating a bleeding angiodysplasia first via injection therapy and then applying APC to achieve hemostatic control, was 179.7 s ± 104.1 s, with 60.9% ± 21.2% treated area, and 27.8% ± 11.5% area of correctly applied intensity. Of the 39 participants included in the evaluation of task two 38 (97.4%) were able to successfully achieve hemostatic control. For the subgroup analysis, participants were split based on their gastroscopy experience into 11 (25%) less experienced and 33 (75%) experienced gastroscopists. The subgroup analysis revealed that experienced gastroscopists required significantly less time to perform the treatment than less experienced gastroscopists for both task one with 65.8 s vs 98.0 s, *p* = 0.008 and task two with 150.4 s vs 254.2 s, *p* = 0.011, as can be seen in Fig. 3. In addition, experienced gastroscopists achieved higher percentages of treated angiodysplasia area than less experienced ones (74.8% vs 65.5%) and slightly higher quality in APC intensity (35.2% vs 32.6%) for task one.

### Usability and workload

**Fig. 4:**
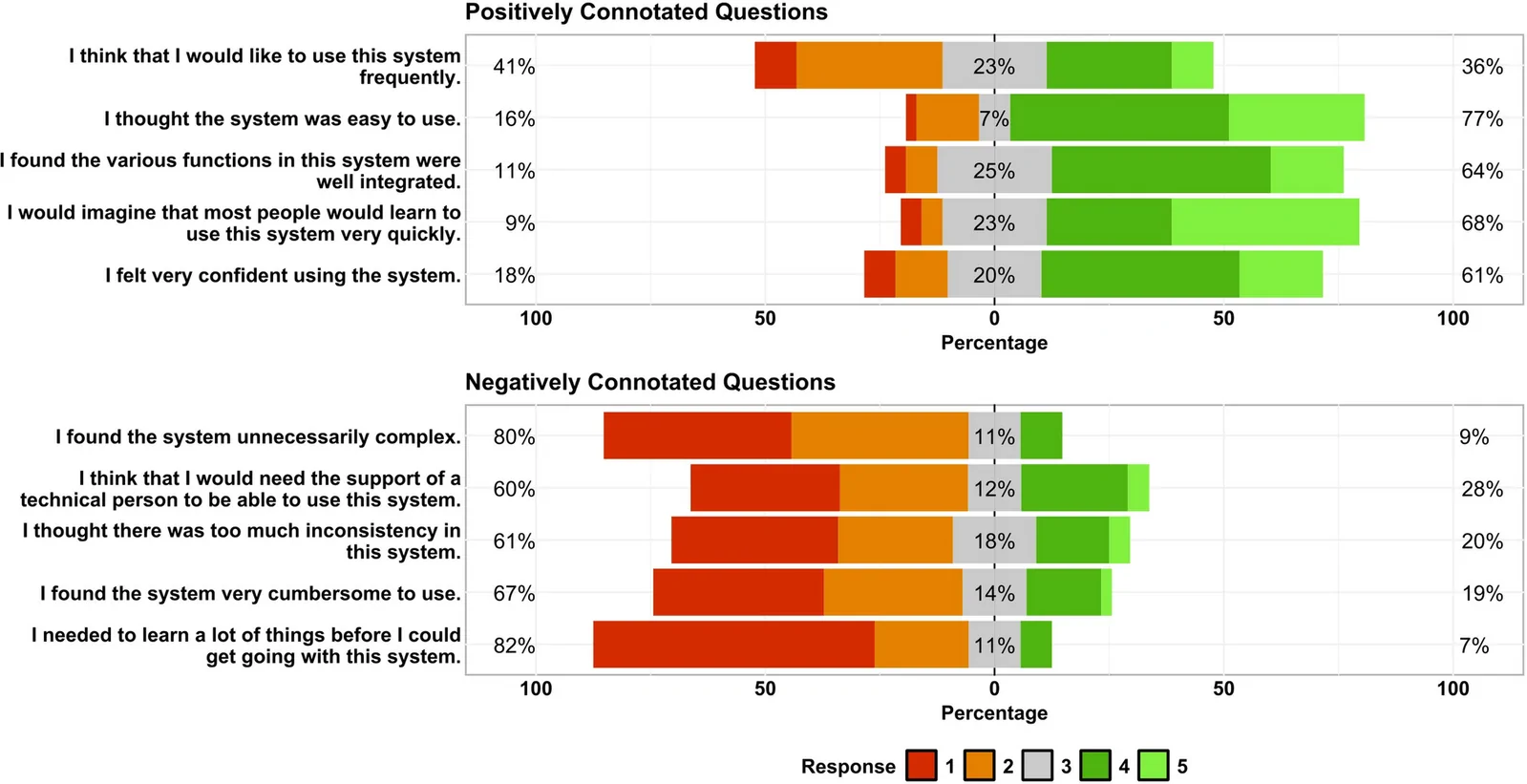
Likert plots of all items from the System Usability Scale. Participants rated the questions on a 5-Point Likert Scale ranging from 1 (strongly disagree) to 5 (strongly agree). For positively connotated questions, higher response values indicate better usability and for negatively connotated questions lower response values indicate better usability

The mean SUS score was 69.6 ± 15.4 (Additional file 4—Sup. Table 1, Sup. Figure 3) and except for the rating of preferring the frequent usage of the simulator with a median of 3 on the SUS scale of 1 (strongly disagree) to 5 (strongly agree) (Q1-Q3 = 2–4), all scores resulted for the positively connotated questions in higher medians of 4 and for all negatively connotated questions in low medians of 1 and 2 (Fig. 4). Furthermore, the mean NASA-TLX score was 34.2 ± 15.1, representing a low workload (Additional file 4—Sup. Figure 4). Participants rated the mental demand 50.5 ± 23.5 and effort 43.3 ± 25.3 with highest values and the temporal demand 21.9 ± 19.7 and frustration 27.4 ± 23.3 with lowest values.

While there was strong agreement among participants, reporting near to no symptoms of cybersickness with a median of 1.0, Q1-Q3 = 1–2 (Additional file 4—Sup. Figure 5), the values for immersion varied for each participant with a median of 3.5, Q1-Q3 = 3–4 (Additional file 4—Sup. Figure 6). For the subgroup analysis, participants were split into groups depending on gender, age, previous VR-experience, immersion, and gastroscopy experience (Additional file 4 – Sup. Table 2). Especially the subgroup analyses for immersion revealed significant differences between low and high immersion. Higher immersion revealed significantly lower NASA-TLX scores (*p* = 0.046), higher SUS scores (*p* = 0.025), and for half of the face validity questions higher realism ratings (*p* < 0.031) (Additional file 4—Sup. Table 3).

### Inductive content analysis

A total of 36 (82%) participants chose to provide optional free-text feedback including improvement suggestions, positive and negative aspects, and further comments. Suggestions, reported with the number of responses from the participants, included adding real-world instruments to control the virtual tools (6) and improving the realism of APC (5), mucosa (4), peristaltic motion (3), injection (3), depth perception (3), and motion artifacts (3). Participants further suggested adding real endoscope footage to the screen (3). In addition, participants appreciated the handling (5) and the approach to virtual endoscopy (4) and evaluated the simulation as a good learning tool (5). Participants also highlighted the artificial feeling of the injection (5) and the unrealistic injected area (3). Five participants further commented that the simulation is great and impressive. A more detailed summary of the inductive content analysis can be found in Additional file 5.

## Discussion

This work introduces OPTESK, a VR training simulator for the treatment of bleeding in the upper GI tract. Simulated scenarios include a non-bleeding and bleeding angiodysplasia treated by injection therapy and APC. OPTESK further uses a real gastroscope as a controller for interaction with the virtual environment. In a user study, OPTESK was evaluated in terms of usability, workload, face, content, and construct validity, immersion, and cybersickness.

Participants agreed (median = 4) that the OPTESK training simulator is a helpful training tool especially for novices and prior to their first intervention on patients. Further, training the application and treatment order of APC and injection using the OPTESK simulator was rated as helpful (median = 4). In addition, the study revealed a shorter task completion time for experienced compared to less experienced endoscopists for task one (65.8 s vs 98.0 s) and task two (150.4 s vs 254.2 s), further suggesting its potential as a training tool for novices to improve their skills. This is consistent with a previous study of Hohlstein et al. (2026) evaluating durations for endoscopic navigational tasks (novices: 85.4 s, beginners: 55.0 s, experienced endoscopists: 47.4 s). Using the OPTESK simulator for novices is further in accordance with a review by Khan et al. (2018) as well as a position statement by the European Society of Gastrointestinal Endoscopy (Antonelli et al. 2024), which encourage validated computer-based simulations as supplement to early conventional endoscopy training. In general, task one was solved faster than task two, which may be due to a greater number of different steps including tool changes, a more challenging position of the lesion, and consequently, more difficult movement of the endoscope to achieve hemostasis.

OPTESK shows a good face validity by achieving high scores especially for the virtual endoscope movement which gets transferred via sensors from the real endoscope, indicating a successful integration of the hardware elements into the VR simulation. Further, the visualization of the APC treatment was rated as highly realistic with a median of 4, comparable to the APC visualization of Erden et al. ( 2024). While some aspects such as the angiodysplasias, stomach texture, and injected areas received moderate realism scores (median = 3–3.5), questions evaluating the content validity such as the helpfulness for training submucosal injection, the simulators’ treatment order, and its potential in training novices received high ratings (median = 4). Conclusively, this raises the question whether highly realistic textures are crucial for the training of UGIB.

Further, OPTESK achieved a mean SUS score of 69.6 comparable to the computer-based endoscopy simulator Endonix (Olympus, Tokyo, Japan) (median = 75) (Hohlstein et al. 2026) and our VR simulator for peri-procedural tasks in colonoscopy ViGaTu (M = 70) (Henniger et al. 2024), indicating good usability (Bangor et al. 2008). All items of the SUS were positively evaluated except for the question of the preference to use the system frequently (median = 3). This may imply, similar to the simulator of Hohlstein et al. (2026), that OPTESK is more suitable for beginner endoscopists. Additionally, similar to Hohlstein et al. (2026), physicians were generally in favor of implementing simulators in endoscopy training (median = 5) and simulator training being mandatory before the first examination on patients (median = 4). In addition, the participants would like to have OPTESK at their own institution (median = 4) and would have it implemented in endoscopy training (median = 4).

The workload while using the simulator, represented by the NASA-TLX scores, achieved low values for subscales like physical and temporal demand and frustration (M = 21.9—27.4). The mental demand and effort were rated as moderate (M = 43.3—50.5), which is consistent with higher cognitive load values from VR simulators in previous work (Hohlstein et al. 2026; Andersen et al. 2023; Pérez-Escamirosa et al. 2023). These findings are associated with stress due to sensory distractions or lack of familiarity with the modality as side effects of full immersion (Mao et al. 2021). Even though stressful and mental demanding environments could cause initially worse performances during training, practicing under duress may hint to greater gains in long-term skills (Mao et al. 2021; Andersen et al. 2023; Bowyer et al. 2008; Sankaranarayanan et al. 2020). Therefore, having moderate values of mental demand and effort, without causing sensory overload and with little frustration may suggest that training applications can benefit by increasing both mental demand and effort to achieve higher context realism and learning effect (Gutiérrez et al. 2007). The results of this study further show that especially the level of immersion had an impact on the participants’ perception of the simulator. Higher immersion led to better general perception of usability, workload, realism, and helpfulness as well as improved scores. This could indicate that higher levels of immersion enhance the training experience and lead to higher knowledge gain (Gutiérrez et al. 2007), due to the perception of being present in a realistic environment with a full magnitude of sensory stimuli (Liu et al. 2023). Based on the above, immersion should be of high priority when developing VR simulators. In addition, the simulator provides the option to further extend and adapt the training environment. For example, inclusion of features such as visual and auditory distractions can be implemented to replicate stressful clinical situations in a controlled environment without consequences for patients and users (Mao et al. 2021; Jensen and Konradsen 2018; Bogar et al. 2024). Further, VR-based training with our simulator may offer a more cost-efficient and scalable alternative to resource consuming training (Mao et al. 2021).

OPTESK follows a do-it-yourself approach, which further supports making the project available to the public. Requirements for the setup include a PC, an endoscope and a VR-headset, which should be readily available to utilize OPTESK. The simulation is made publicly available, alongside thorough documentation on how to install it. The 3D blueprints for printing the hardware components are also made available, and can be printed with any low cost, commercially available 3D printer and material. Regarding the sensor setup, the sensors are low cost, and the extensive instructions and cabling diagram can be used to implement the electronic assembly with minimal knowledge of electronics.

This work also faces limitations that identify new directions for future work. While participants were recruited at two large national congresses which could introduce selection bias, the participants’ demographics showed a wide range of experiences and characteristics. Furthermore, conducting the study at congresses could have influenced the perception of the simulator due to the uncommon environment or time restrictions. However, participation was voluntary during multiple days throughout the conferences. Further, in the current implementation, the endoscope moves linearly and only rotations are tracked, which results in a limited range of maneuvers that can be performed during inspection. However, OPTESK focuses on lesion treatment using APC and injection therapy in a realistic virtual environment, which can be achieved with the motions that our setup covers. The current version of OPTESK simulates only non-bleeding and bleeding angiodysplasias within the fundus of the stomach, which are treated by injection therapy and APC. Future work will introduce new pathologies and tools for training the treatment of UGIB. Also, future work should compare immersive to non-immersive VR training simulators to investigate the advantages of each approach, ideally with performance improvement in real clinical cases as primary outcome. The simulator can be enhanced by adding new pathologies, gamification elements (Turchet et al. 2025), and artificial intelligence assistance (Bogar et al. 2024), and placing them in different parts of the upper GI tract.

## Conclusions

This paper introduced OPTESK, an open-source VR training simulator for emergency management in the upper GI tract, by integrating a real endoscope for interaction with the virtual environment. Experts found OPTESK to be a useful training tool, particularly due to its good usability and content and construct validity, with different task durations for experienced and less experienced gastroscopists.

## Supplementary Information

Additional file 1. OPTESK VR Simulator - Hardware Setup. Description and figure of the hardware setup.

Additional file 2. Non-Standardized Questionnaires. List of all non-standardized questionnaires developed for the study.

Additional file 3. Missing Data Management. Description of the missing data management.

Additional file 4. Additional Figures and Descriptive Statistics from the Analysis. Supplementary figures, tables and descriptions from the analysis.

Additional file 5. Inductive Content Analysis. Detailed inductive content analysis of the free-text feedback of the post-questionnaire.

## Data Availability

The complete hardware setup including 3D printing files, schematics of the wiring, and the software are available in the OPTESK_Supplementary_data public repository, https://share.ukw.de/index.php/s/3ttXgJajntqcDiY.

## Abbreviations

3D: Three-dimensional
APC: Argon Plasma Coagulation
BMFTR: Bundesministerium für Forschung, Technologie und Raumfahrt (Federal Ministry of Research, Technology and Space)
CPU: Central Processing Unit
DGE-BV: Deutsche Gesellschaft für Endoskopie und Bildgebende Verfahren e.V. (German Society for Endoscopy and Imaging Techniques)
DGIM: Deutsche Gesellschaft für Innere Medizin (German Society of Internal Medicine)
ESD: Endoscopic Submucosal Dissection
GI: Gastrointestinal
GPU: Graphics Processing Unit
HMD: Head-Mounted Display
IMU: Inertial Measurement Unit
M: Mean
NASA: National Aeronautics and Space Administration
NASA-TLX: NASA Task Load Index
OPTESK: Offene Plattform zur Tiermaterialfreien Endoskopischen Simulation und Komplikationsmanagement (Open platform for animal material-free endoscopic simulation and complication management)
Q1-Q3: First to Third Quartile
SD: Standard Deviation
SUS: System Usability Scale
UGIB: Upper Gastrointestinal Bleeding
ViGaTu: Virtual Gastro Tutor
VR: Virtual Reality
