# Supplementary material for: Evaluation of the OPTESK VR training simulator for gastric bleeding treatment
Source: Bioelectron Med. 2026 Oct 19;12:22. doi: 10.1186/s42234-026-00216-w (PMC13681957; doi:10.1186/s42234-026-00216-w)
Supplement: Supplementary file 1 [file 42234_2026_216_MOESM1_ESM.pdf]

OPTESK VR Simulator - Hardware Setup

The central element of the simulator's hardware is an ESP32-C3 breakout board (AZ-Delivery Vertriebs GmbH, Deggendorf, Germany), which is a microcontroller with inbuilt Wi-Fi and Bluetooth, enabling wireless transmission of data. A BNO055 Intelligent 9-Axis Absolute Orientation Sensor (Adafruit Industries, LLC, United States) is embedded at the beginning of the controllable part of the endoscope tip, as shown in **Sup. Figure 1**, outputting unit quaternions values at 100Hz to map pitch (up/down), yaw (left/right), and roll (rotation). This is providing a 5 Degrees-of-freedom tracking of the bending tip of the endoscope. The endoscope chord is connected to a linear slider mechanism which allows to enable and track pushing the endoscope forwards and pulling it backwards. A VL53L0X Time-of-Flight Infrared distance sensor (STMicroelectronics, Geneva, Switzerland) tracks insertion depth (sliding motion) by measuring how much the slider moved forward/backward from its initial position. Additionally, the system provides two pedals which serve as trigger inputs for APC, injection and waterjet activation. The data transmission from the Esp32-C3 was established using serial communication. Mechanical holders for all the assemblies were designed using Fusion 360 (Autodesk Inc., San Francisco, California, United States) and were 3D printed with the Bambulabs-PS1 3D printer (Bambu lab, Shenzhen, China) using Polylactic Acid filament.

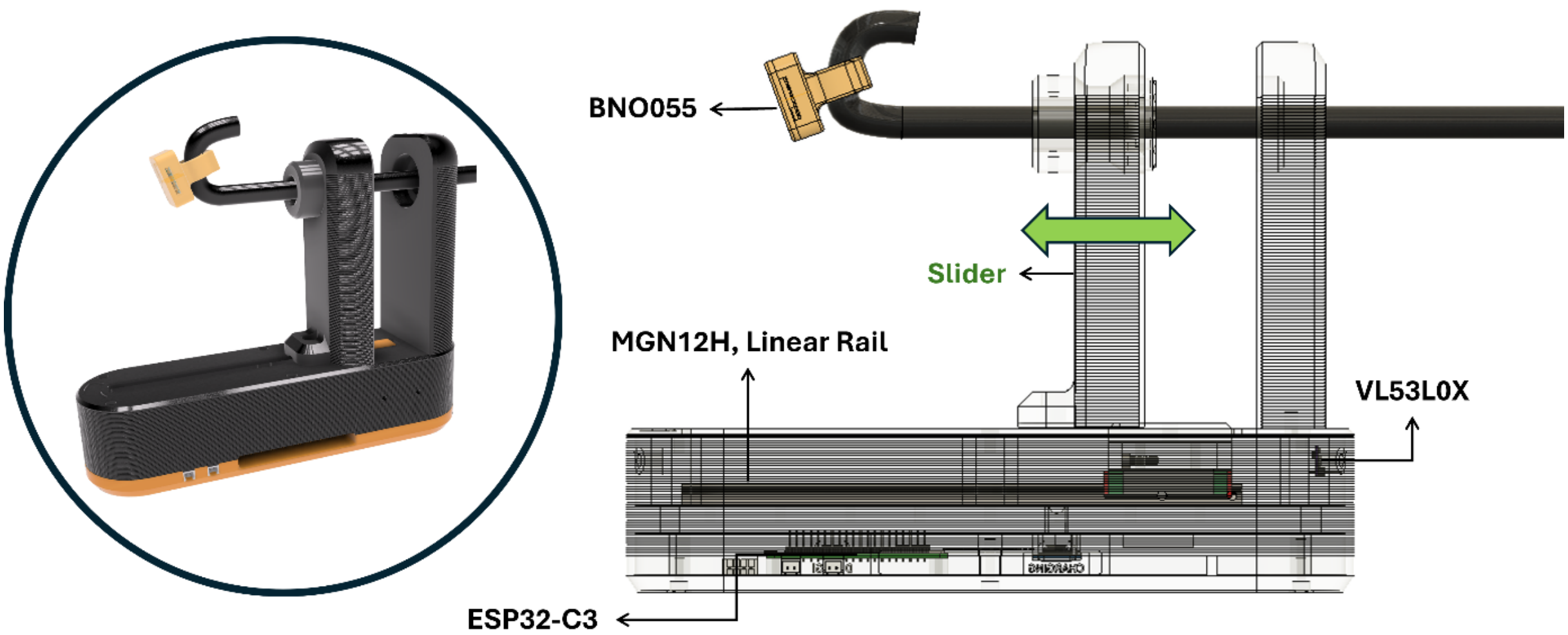

Supplementary Figure 1: Mechanical model and detailed component breakdown of the endoscope holder and the integrated sensory modules.
