# Supplementary material for: Evaluation of the OPTESK VR training simulator for gastric bleeding treatment
Source: Bioelectron Med. 2026 Oct 19;12:22. doi: 10.1186/s42234-026-00216-w (PMC13681957; doi:10.1186/s42234-026-00216-w)
Supplement: Supplementary file 2 [file 42234_2026_216_MOESM2_ESM.pdf]

Non-Standardized Questionnaires

	strongly disagree				*strongly agree*
**Simulators & VR-Experience**	**1**	**2**	**3**	**4**	**5**
Simulator training in general (any simulator) should be implemented in endoscopy training.	O	O	O	O	O
Simulator training (any simulator) should be mandatory before starting endoscopy in patients.	O	O	O	O	O
I have experience with virtual reality systems (VR-glasses).	O	O	O	O	O

	none				**severe**
**Cybersickness**	**1**	**2**	**3**	**4**	**5**
How severe did you experience symptoms of cybersickness (e.g., nausea, dizziness, general discomfort) during the VR simulation?	O	O	O	O	O

	strongly disagree				*strongly agree*
**Immersion**	**1**	**2**	**3**	**4**	**5**
I felt present in the virtual environment, as if I was really there.	O	O	O	O	O

	strongly disagree				*strongly disagree*
**Face Validity**	**1**	**2**	**3**	**4**	**5**
The stomach texture is realistically represented.	O	O	O	O	O
The angiodysplasias are realistically represented.	O	O	O	O	O
The stomach anatomy is realistically represented.	O	O	O	O	O
The endoscope footage (regarding light, distortion, color) is realistically represented.	O	O	O	O	O
The virtual camera moves according to the operation of the real endoscope.	O	O	O	O	O
The injected areas within the stomach are realistically represented.	O	O	O	O	O
The application of injection is realistically represented.	O	O	O	O	O

The APC treated areas within the stomach are realistically represented.	O	O	O	O	O
The application of APC is realistically represented.	O	O	O	O	O
The interaction with the stomach wall is realistically represented.	O	O	O	O	O
The tools (APC, Needle) are realistically represented.	O	O	O	O	O
All in all, the simulator was realistic.	O	O	O	O	O

	strongly disagree				***strongly agree***
**Content Validity**	**1**	**2**	**3**	**4**	**5**
The simulator is helpful to train the application of APC.	O	O	O	O	O
The simulator is helpful to train the application of injection.	O	O	O	O	O
The simulator is helpful to train the order of treatment with adrenaline and APC.	O	O	O	O	O
The simulator is helpful to train novices before patient endoscopy.	O	O	O	O	O
The simulator is a helpful training tool for novices.	O	O	O	O	O
The simulator is a helpful training tool for experts.	O	O	O	O	O
After testing the simulator, should it be implemented in endoscopy training?	O	O	O	O	O
Would you like to have the simulator at your institution?	O	O	O	O	O

	No, not at all				***Yes, very helpful***
**Content Validity**	**1**	**2**	**3**	**4**	**5**
Do you find the scores and graphs for various parameters helpful for improving your own skills?	O	O	O	O	O
