# Supplementary material for: Evaluation of the OPTESK VR training simulator for gastric bleeding treatment
Source: Bioelectron Med. 2026 Oct 19;12:22. doi: 10.1186/s42234-026-00216-w (PMC13681957; doi:10.1186/s42234-026-00216-w)
Supplement: Supplementary file 3 [file 42234_2026_216_MOESM3_ESM.pdf]

Missing Data Management

One participant was excluded from the evaluation of the task durations and scores for both tasks because of missing data and four participants were excluded only from task two because of not finishing task two due to time constraints from session schedules at the conference. Because of missing data, one participant was excluded for the evaluation of all content validity questions and one for the evaluation of the combined SUS score. For one participant, the NASA-TLX score was calculated without the mental demand. Additional missing values included one value for the realism of the stomach anatomy, two for the realism of the application of injection, and one further for the question whether they would like to have the simulator at their institution and if the scores were helpful to increase their own skills.
