# Supplementary material for: Evaluation of the OPTESK VR training simulator for gastric bleeding treatment
Source: Bioelectron Med. 2026 Oct 19;12:22. doi: 10.1186/s42234-026-00216-w (PMC13681957; doi:10.1186/s42234-026-00216-w)
Supplement: Supplementary file 4 [file 42234_2026_216_MOESM4_ESM.pdf]

Additional Figures and Descriptive Statistics from the Analysis

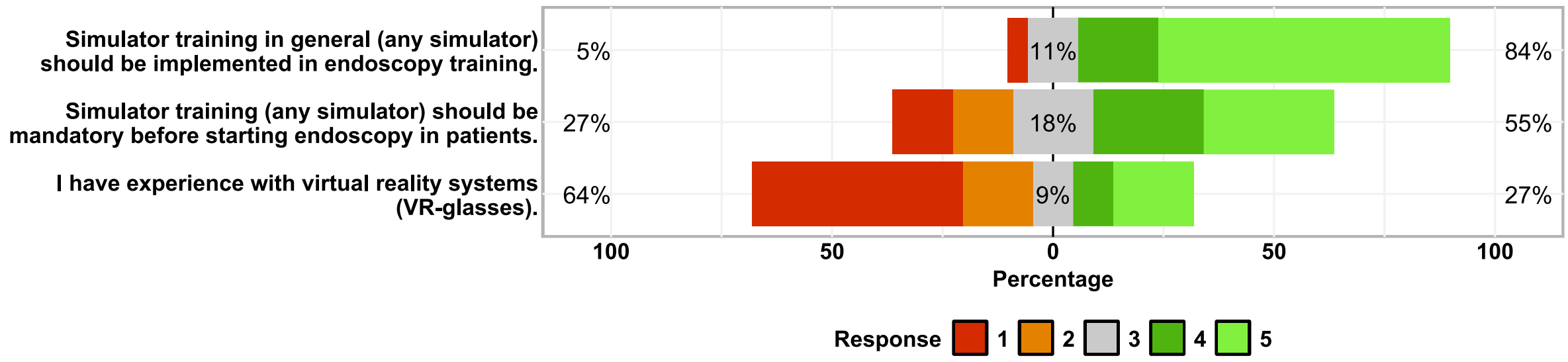

Supplementary Figure 1: Likert plots of the general opinions on simulator training and VR-experience. Participants rated the questions on a 5-Point Likert Scale ranging from 1 (strongly disagree) to 5 (strongly agree).

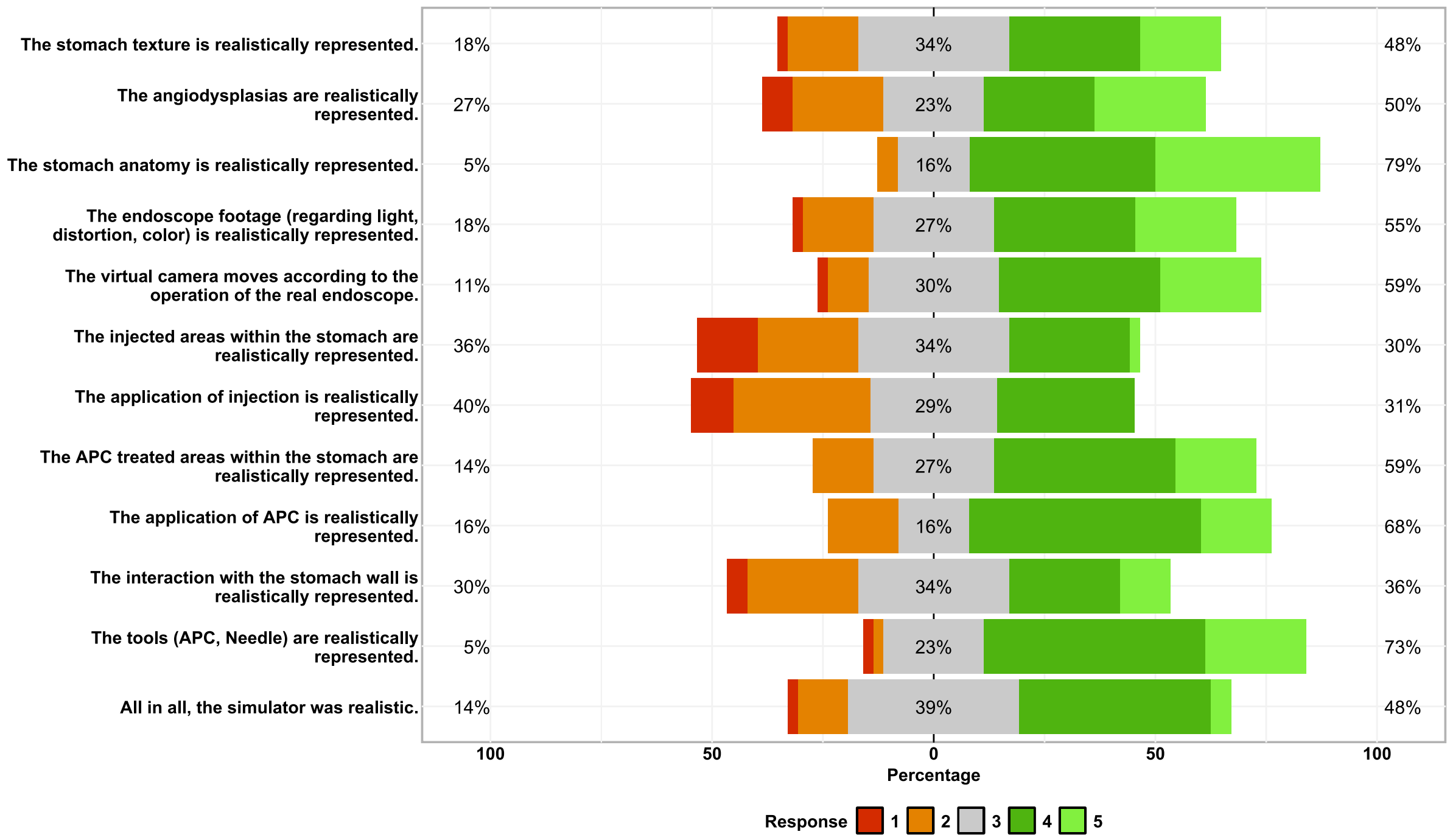

Supplementary Figure 2: Likert plots of the face validity questions. Participants rated the questions on a 5-Point Likert Scale ranging from 1 (strongly disagree) to 5 (strongly agree).

Participants agreed with a median of 4, that the VR simulator's virtual gastroscope movement (Q1-Q3 = 3-4), visualization (Q1-Q3 = 3-4) and application (Q1-Q3 = 3-4) of APC, instruments (Q1-Q3 = 3-4), stomach anatomy (Q1-Q3 = 4-5), and endoscopy screen footage (Q1-Q3 = 3-4) were realistic (**Sup. Figure 2**). Participants rated the realism of the angiodysplasias with a median of 3.5 (Q1-Q3 = 2-4.25), and the stomach texture (Q1-Q3 = 3-4), injected areas (Q1-Q3

= 2-4), the application of injection (Q1-Q3 = 2-4), the interaction with the stomach wall (Q1-Q3 = 2-4), as well as the general realism (Q1-Q3 = 3-4) with a median of 3.

Supplementary Table 1: Descriptive Statistics of the SUS reported with medians and first and third quartiles.

**Positively Connotated Questions**	**Median (Q1 - Q3)**
I think that I would like to use this system frequently.	3.0 (2.0 -4.0)
I thought the system was easy to use.	4.0 (4.0 - 5.0)
I found the various functions in this system were well integrated.	4.0 (3.0 - 4.0)
I would imagine that most people would learn to use this system very quickly.	4.0 (3.0 - 5.0)
I felt very confident using the system.	4.0 (3.0 - 4.0)
**Negatively Connotated Questions**	**Median (Q1 - Q3)**
I found the system unnecessarily complex.	2.0 (1.0 - 2.0)
I think that I would need the support of a technical person to be able to use this system.	2.0 (1.0 - 4.0)
I thought there was too much inconsistency in this system.	2.0 (1.0 - 3.0)
I found the system very cumbersome to use.	2.0 (1.0 - 3.0)
I needed to learn a lot of things before I could get going with this system.	1.0 (1.0 - 2.0)

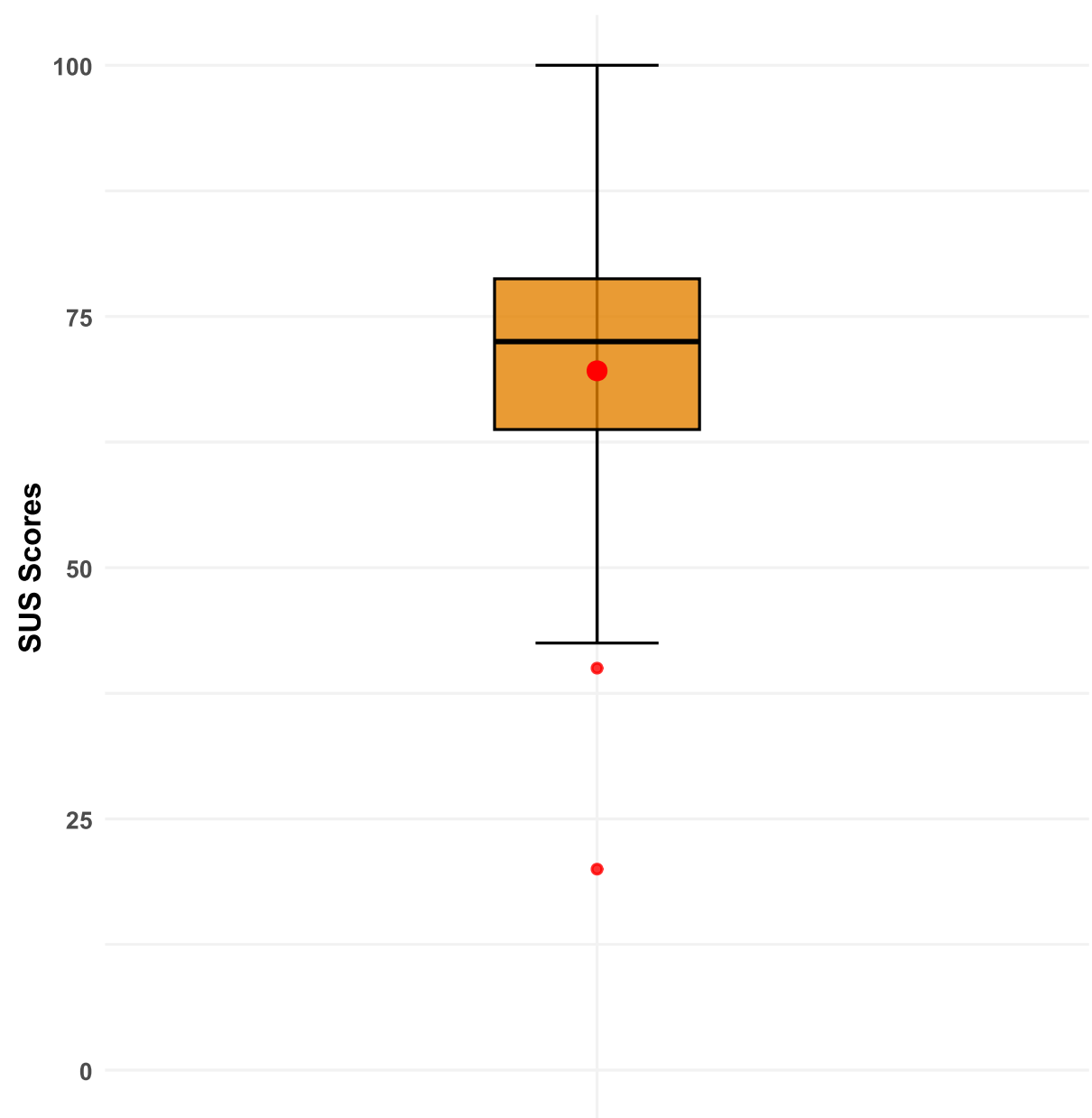

Supplementary Figure 3: Box-and-whisker plot of the combined SUS scores, ranging from 0 (worst imaginable) to 100 (best imaginable) usability. Whiskers denote the 1.5 × interquartile range and points beyond the whiskers are plotted as outliers.

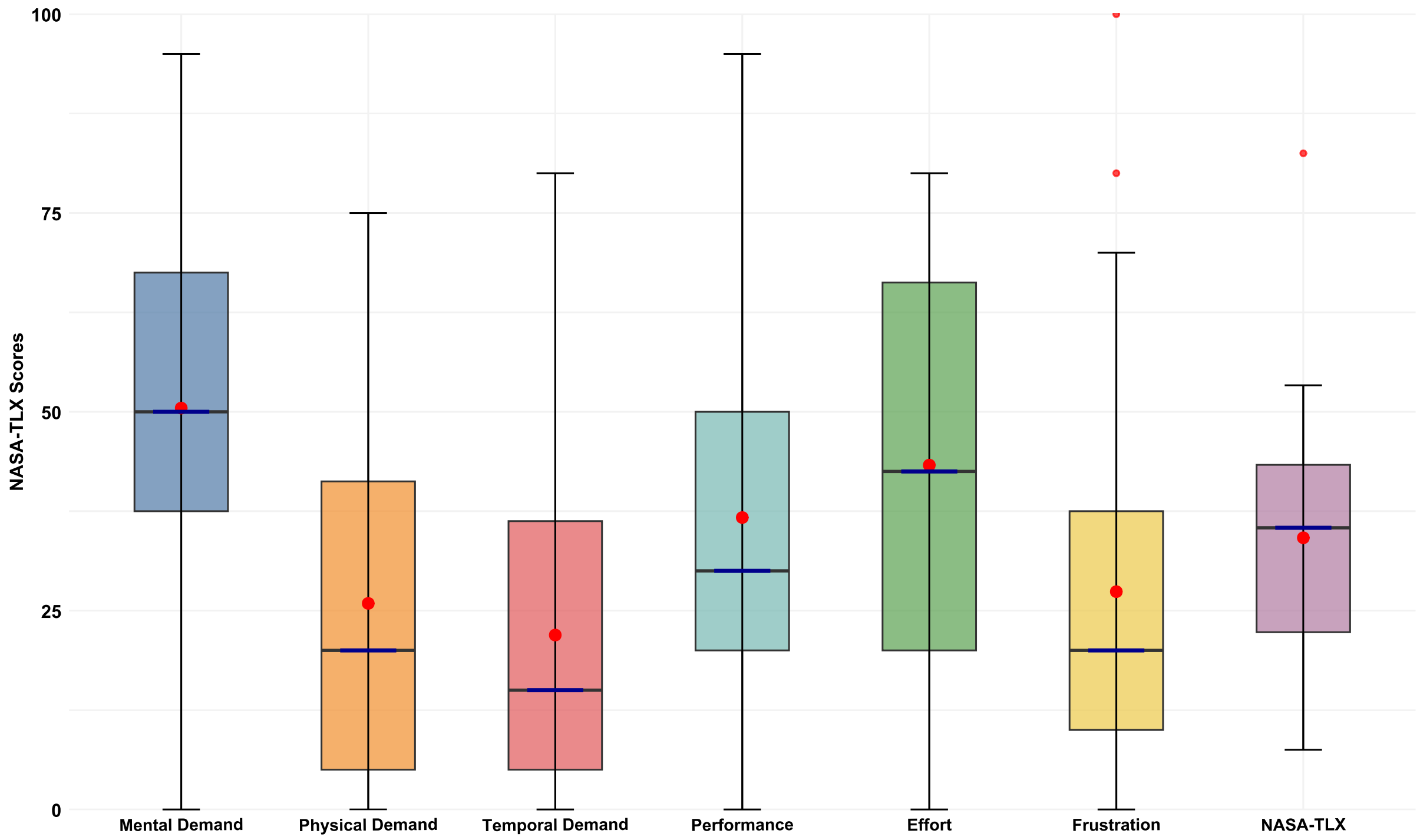

Supplementary Figure 4: Box-and-whisker plots of the combined NASA-TLX scores and its subscales, ranging from 0 (very low) to 100 (very high). Whiskers denote the 1.5 × interquartile range and points beyond the whiskers are plotted as outliers.

The mean NASA-TLX score was 34.2 ± 15.1 (**Sup. Figure 4**). The mean mental demand was rated with 50.5 ± 23.5, the effort with 43.3 ± 25.3, and the performance with 36.7 ± 24.7. Furthermore, participants rated the physical demand with a mean of 25.9 ± 23.6, the temporal demand with 21.9 ± 19.7, and the frustration with 27.4 ± 23.3.

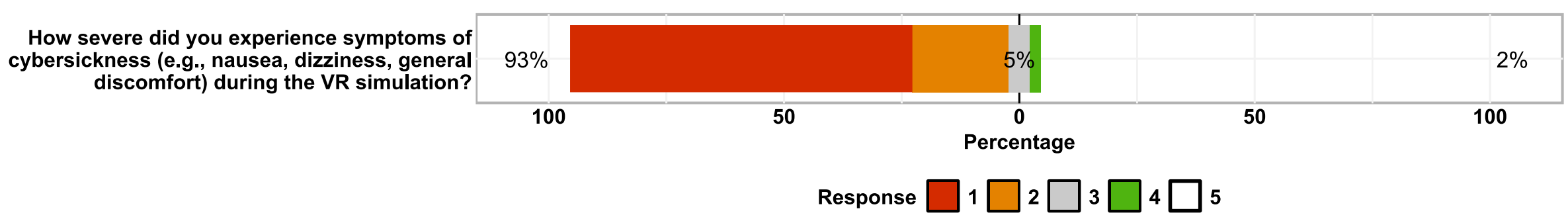

Supplementary Figure 5: Likert plot of cybersickness. Participants rated the questions on a 5-Point Likert Scale ranging from 1 (none) to 5 (severe).

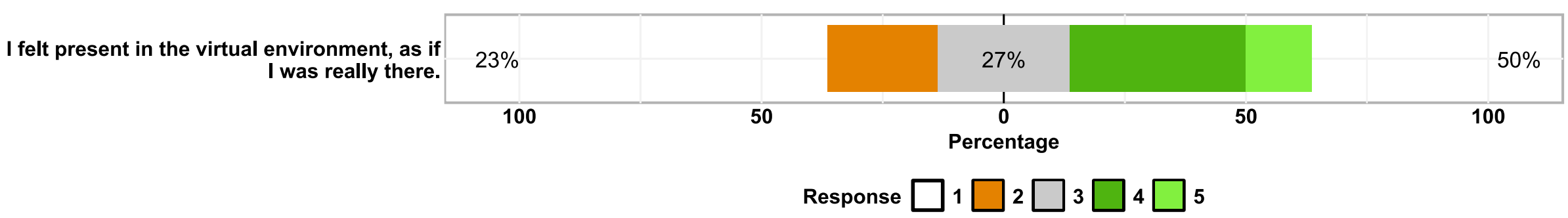

Supplementary Figure 6: Likert plot of immersion. Participants rated the questions on a 5-Point Likert Scale ranging from 1 (strongly disagree) to 5 (strongly agree).

Supplementary Table 2: Distribution of participants across subgroups.

Characteristic	Value
Gender, n (%)	
Male	30 (68.2%)
Female	14 (31.8%)
Age, n (%)	
Aged ≤ 40	22 (50%)
Aged > 40	22 (50%)
VR-Experience, n (%)	
No VR-Experience (=1)	21 (47.7%)
VR-Experience (>1)	23 (52.3%)
Immersion, n (%)	
Low Immersion (≤3)	22 (50%)
High Immersion (>3)	22 (50%)
Gastroscopy Experience, n (%)	
Less Experience (≤ 500 performed gastroscopies)	11 (25%)
Experience (> 500 performed gastroscopies)	33 (75%)

Supplementary Table 3: Significant results of the statistical analysis. Reported are either means (M) or medians and p-values.

Gastroscopy-Experience	Experienced vs Less Experienced	p-Value
Task durations task one	M = 65.8s vs M = 98.0s,	$p = .008$
Task durations task two	M = 150.4s vs M = 254.2s,	$p = .011$
**Immersion**	**High Immersion vs Low Immersion**	
NASA-TLX	M = 29.6 vs 38.7	$p = .046$
Temporal demand	M = 13.4 vs 30.5	$p = .006$
Frustration	M = 19.3 vs 35.5	$p = .048$
Effort	M = 32.3 vs 54.3	$p = .002$
SUS	M = 74.7 vs 64.3	$p = .025$
System complexity	Median = 1.0 vs 2.0	$p = .046$
Ease of use	Median = 4.0 vs 4.0	$p = .021$
Easiness to learn the system	Median = 5.0 vs 4.0	$p = .004$
Amount to learn prior to using the system	Median = 1.0 vs 2.0	$p = .016$
Realism stomach texture	Median = 4.0 vs 3.0	$p = .011$
Realism endoscopy screen footage	Median = 4.0 vs 3.0	$p = .004$
Realism injected areas	Median = 3.0 vs 2.5	$p = .017$
Realism application of APC	Median = 4.0 vs 3.5	$p = .031$
Realism interaction with stomach wall	Median = 4.0 vs 2.5	$p < .001$
General realism	Median = 4.0 vs 3.0	$p = .001$
Helpful for training APC	Median = 4.0 vs 4.0	$p = .040$
**VR-Experience**	**no VR-experience vs VR-experience**	
Task durations task two	M = 145.5s vs 209.0s	$p = .049$
**Gender**	**Female vs Male**	
Frustration	M = 36.8 vs 23.0	$p = .028$
Realism treated APC areas	Median = 4.0 vs 3.5	$p = .033$
