# Supplementary material for: Evaluation of the OPTESK VR training simulator for gastric bleeding treatment
Source: Bioelectron Med. 2026 Oct 19;12:22. doi: 10.1186/s42234-026-00216-w (PMC13681957; doi:10.1186/s42234-026-00216-w)
Supplement: Supplementary file 5 [file 42234_2026_216_MOESM5_ESM.pdf]

Inductive Content Analysis

At the end of the questionnaire, participants were given the option to provide additional free-text feedback on suggestions to improve the simulator, positive and negative aspects, as well as further comments. 36 out of 44 participants (82%) chose to provide responses for some of the questions. Participants were also able to include multiple aspects for each question. The feedback was documented and summarized in categories. For reporting, we used a cutoff of ≥ 2 responses for a category to focus on the aspects with recurrence across the participants. From 33 participant responses resulting in 40 aspects, most suggestions to improve the system, included a real world instrument for pushing and pulling and manipulating the virtual APC and Injection tool (6), to improve the realism of APC (5), the mucosa (4), the peristaltic (3), injection (3), depth perception (3), motion artifacts (3), and interaction with the stomach wall (2). A suggestion for future development was to display real endoscope footage on the screen (3). Regarding positive aspects from 31 participants resulting in 40 aspects, participants liked the handling (5) and appraised the simulation as a good learning tool (5) and approach to virtual endoscopy (4). Further positive aspects included APC (2), the realism (3), the stomach anatomy (2), the angiodysplasias (2), the endoscope movement (2), the realistic images (2), the haptic feeling due to the real endoscope (2), and the implementation of the hardware parts (2). Regarding the negative aspects 23 participants also gave 24 aspects including an artificial feeling from injection (5), visual unrealistic injected area (3), the need for better task descriptions and guidelines (2), the virtual buttons and controllers (2), as well as the position of the virtual and real endoscope tip not matching (2). Regarding further comments, five participants emphasized that the simulation is great and impressive work.
